# Relationship between ABO blood group and gestational diabetes mellitus: A protocol for systematic review and meta-analysis

**DOI:** 10.1097/MD.0000000000025877

**Published:** 2021-05-14

**Authors:** Dongyun Chen, Lili Lin, Qiong Hong, Xiaohong Li

**Affiliations:** a Department of Infection Management; b Department of Obstetrics and Gynecology; c Department of Ultrasound, Ruian Maternity and Child Care Hospital, Ruian, Zhejiang province, China.

**Keywords:** ABO blood group, gestational diabetes mellitus, meta-analysis, protocol

## Abstract

**Background:**

Gestational diabetes mellitus (GDM) is a common metabolic disorder syndrome in women during pregnancy. If effective measures are not taken to intervene in the early stage of GDM, severe effects will damage maternal and infant health. ABO is the most important human blood group system. A large number of studies have displayed that ABO blood group is associated with many diseases. At present, the risk relationship between ABO blood group and GDM is controversial. The purpose of this study is to explore the risk relationship between ABO blood group and GDM by meta-analysis, thus providing basis for the prevention and treatment of GDM.

**Methods:**

An electronic database, including Embase, Cochrane Library, Pubmed, Chinese databases SinoMed, Chinese National Knowledge Infrastructure, Chinese Scientific Journals Database and Wanfang Data, will be used to search for studies of ABO blood group and GDM. The language will be limited to Chinese and English. The two reviewers will be responsible for the selection of the study, the extraction of data and the evaluation of the quality of the research. All statistical analyses will be carried out using Review Manager 5.3.

**Results:**

The results of this meta-analysis will be published in peer-reviewed journals.

**Conclusion:**

This study will provide evidence to support the relationship between ABO blood group and the risk of GDM

**Ethics and dissemination:**

The private information from individuals will not be published. This systematic review also will not involve endangering participant rights. Ethical approval is not required. The results may be published in a peer-reviewed journal or disseminated in relevant conferences.

**OSF Registration number:**

DOI 10.17605/OSF.IO/W6QSX.

## 1 Introduction

In addition to type 1 diabetes and type 2 diabetes, gestational diabetes mellitus (GDM) is another kind of diabetes.^[1]^ It is a common metabolic disorder syndrome in women during pregnancy.^[2]^ The diagnosis is generally based on the results of oral glucose tolerance test in the second trimester of pregnancy. If effective measures are not taken to intervene in the early stage of GDM, severe effects will damage maternal and infant health. GDM increases various risks, such as preeclampsia, fetal death, fetal malformation, macrosomia, dystocia and cesarean section.^[3–6]^ Women suffering from GDM also had a higher risk of diabetes within 10 years after delivery.^[5]^ The incidence of GDM in women of childbearing age has increased due to the continuous prevalence of obesity and diabetes in the past decade.^[7]^ Therefore, early monitoring of the risk of GDM is of great significance to maternal and infant health.

ABO is the most important human blood group system.^[8]^ In general, human blood type does not change from the time of embryo formation. Many studies have revealed that ABO blood group is related to infection, cancer, cardiovascular disease, nervous system disease, and so on.^[9–13]^ Previous studies have also explored the relationship between ABO blood group and pregnancy complications.^[14]^ However, the potential link between ABO blood type and adverse pregnancy outcomes is controversial, including preeclampsia and related diseases, venous thromboembolism, postpartum hemorrhage, and GDM.

ABO antigens influence several biomarkers and is closely related to insulin resistance and the development of type 2 diabetes, such as E-selectin, P-selectin, tumor necrosis factor-α, soluble intercellular adhesion molecule-1 and interleukin-6.^[15,16]^ Some studies have explored the relationship between ABO blood type and diabetes, but the findings are mostly inconsistent.^[14,17,18]^ However, most of the studies on ABO blood group and its relationship with GDM are also inconsistent.^[14]^

Although it has been reported that AB blood type is a protective risk factor of GDM,^[19]^ some studies have reported that AB blood type increases the risk of GDM.^[20,21]^ On the other hand, in a study carried out in Thailand, there was no relationship between ABO blood group and GDM.^[22]^ To date, the evidence for the relationship between ABO blood group and GDM is still limited and inconsistent. We conducted a meta-analysis to further confirm the relationship between ABO blood group and GDM.

## 2 Methods

### 2.1 Study registration

The protocol was registered in Open Science Framework (OSF) (registration number: DOI 10.17605/OSF.IO/W6QSX). It was reported that this systematic review and meta-analysis are in conformed with the preferred reporting items for systematic reviews and meta-analysis protocols (PRISMA-P) 2015.^[23]^

### 2.2 Ethic

The review does not involve the assessment of patients’ individual information or rights, so there is no need to obtain approval from an ethical institution.

### 2.3 Inclusion criteria

Studies would be included in this meta-analysis based on following criteria:

1) Study types: All studies related to ABO blood type and GDM susceptibility should be included.
2) Participant type: GDM should be included in the meta-analysis.
3) Outcome: GDM risk comparisons.

### 2.4 Exclusion criteria

According to the following criteria, studies should be excluded from the meta-analysis: conference summaries, incomplete data studies, repeated published studies, and case series.

### 2.5 Search strategy

Embase, Cochrane Library, Pubmed, Chinese databases SinoMed, Chinese National Knowledge Infrastructure (CNKI), Chinese Scientific Journals Database (VIP) and Wanfang Data were searched. The details of PubMed's search strategy are illustrated in Table 1, including all search terms, while similar search strategies are applied to other electronic databases.

**Table 1:** Search strategy in PubMed database.

Number	Search terms
#1	Diabetes, Gestational [MeSH]
#2	Diabetes Mellitus, Gestational [Title/Abstract]
#3	Diabetes, Pregnancy-Induced [Title/Abstract]
#4	Gestational Diabetes [Title/Abstract]
#5	Diabetes, Pregnancy Induced [Title/Abstract]
#6	Gestational Diabetes Mellitus [Title/Abstract]
#7	Pregnancy-Induced Diabetes [Title/Abstract]
#8	or/1–7
#9	ABO Blood-Group System [MeSH]
#10	ABH Blood Group [Title/Abstract]
#11	ABO Factors [Title/Abstract]
#12	Blood Group H Type 1 Antigen [Title/Abstract]
#13	H Blood Group [Title/Abstract]
#14	H Blood Group System [Title/Abstract]
#15	ABH Blood Groups [Title/Abstract]
#16	ABO Blood Group System [Title/Abstract]
#17	ABO Blood-Group Systems [Title/Abstract]
#18	ABO Factor [Title/Abstract]
#19	Blood Group, ABH [Title/Abstract]
#20	Blood Group, H [Title/Abstract]
#21	Blood Groups, ABH [Title/Abstract]
#22	Blood Groups, H [Title/Abstract]
#23	Blood-Group System, ABO [Title/Abstract]
#24	Blood-Group Systems, ABO [Title/Abstract]
#25	Factor, ABO [Title/Abstract]
#26	Factors, ABO [Title/Abstract]
#27	H Blood Groups [Title/Abstract]
#28	System, ABO Blood-Group [Title/Abstract]
#29	Systems, ABO Blood-Group [Title/Abstract]
#30	or/9-29
#31	#8 and #30

### 2.6 Data collection and analysis

#### 2.6.1 Selection of studies

**Figure 1:**
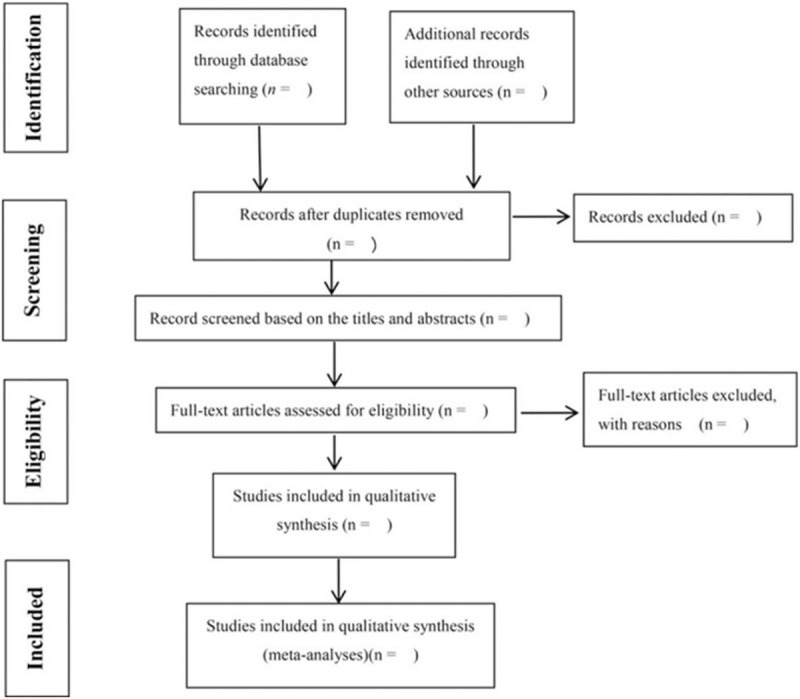
Flow diagram of study selection process.

Two researchers independently complete the literature screening, exclude the studies that obviously do not meet the inclusion criteria, and further read the abstracts and the full texts, to determine whether they meet the inclusion criteria. The data included in the literature will be extracted and cross-checked. Disagreement should be solved by consulting a third researcher, thus reaching a consensus. The screening flow chart of this study is displayed in Figure 1.

#### 2.6.2 Data extraction

We extracted data from literatures that are conformed to the meta-analysis. The data include the first author, year of publication, country, race of each study population, number of cases and controls, average age, ABO blood type and number of cases of GDM, etc.

#### 2.6.3 Study quality assessment

Two researchers independently applied Newcastle-Ottawa scale (NOS) to evaluate literatures included in the analysis, and adopted third-party opinions if there exist any differences.^[24,25]^ Literatures with a total score of more than 6 are considered to be high quality.

#### 2.6.4 Dealing with missing data

The research focuses on the defects of the original data. We contacted the author by email to ask for the original data. If the original data is not available, then we would analyze the existing data.

### 2.7 Statistical analysis

A meta-analysis was performed using RevMan 5.3 (provided by Cochrane Collaboration). Odds ratio (OR) is the statistic of effect analysis, and each effect dose provides 95% of confidence interval (CI). Heterogeneity was ascertained using I^2^. I^2^ < 50% revealed that the studies exhibited homogeneity, so fixed effects model was adopted. Otherwise, the random effects model was adopted. In the presence of heterogeneity, sensitivity analyses and subgroup analysis would be conducted to investigate the heterogeneity sources.

### 2.8 Subgroup analysis

According to patient race, sample size, history of diabetes, and so on, we carried out subgroup analysis.

### 2.9 Sensitivity analysis

In order to test the stability of meta-analysis results of indicators, a one-by-one elimination method will be adopted for sensitivity analysis.

### 2.10 Assessment of publication biases

If more than 10 studies are included, a funnel chart would be applied to assess the report bias.^[26,27]^

## 3 Discussion

As a common disease, GDM is defined as various degrees of impaired glucose tolerance due to seizures or first recognition during pregnancy, thus affecting about 5 per cent of pregnancies worldwide. GDM is not only associated with adverse pregnancy outcomes, such as macrosomia, dystocia, birth trauma and neonatal metabolic complications, but also with a powerful predictor of postpartum transition to obvious diabetes.^[21]^ The incidence of GDM ranges from 2% to 14%, and the incidence is as high as 40% among obese people. With the increase of the obesity rate of women of childbearing age at this stage, the incidence of GDM is increasing day by day.^[28]^

The association between ABO blood group and DM has been observed in a number of epidemiological and genetic studies, thereby resulting in inconsistent findings. However, in the literature, the researches on the relationship between ABO blood group and GDM^[5]^ are still insufficient. The red blood cell count of pregnant women with AB blood group was significantly higher than that of pregnant women with A and O blood groups, and the levels of fasting blood glucose, urea and serum creatinine of pregnant women with AB blood group were remarkably higher than those of pregnant women with A blood group.^[29]^ These results indicated that ABO blood type may be associated with some adverse pregnancy outcomes. Karagoz et al discovered that patients with AB and O blood groups had a higher risk of gestational diabetes.^[21]^ Shimodaira proposed that AB blood type is a risk factor for GDM.^[30]^ Zhang et al put forward that women with type B or O blood groups were associated with the increased risk of GDM, and type AB blood was a protective factor for GDM in pregnant women.^[19]^ Therefore, in view of the inconsistency of the above results. In this study, we systematically evaluate the risk relationship between ABO blood group and GDM, to provide evidence-based medicine for future clinical guidance.

## Author contributions

**Conceptualization:** Xiaohong Li, Dongyun Chen.

**Data curation:** Dongyun Chen.

**Funding acquisition:** Xiaohong Li.

**Investigation:** Dongyun Chen.

**Project administration:** Xiaohong Li.

**Resources:** Dongyun Chen.

**Software:** Lili Lin.

**Validation:** Lili Lin, Qiong Hong.

**Visualization:** Lili Lin, Qiong Hong.

**Writing – original draft:** Xiaohong Li, Dongyun Chen.

**Writing – review & editing:** Xiaohong Li, Dongyun Chen.

## References

[1] Association AD. Diagnosis and classification of diabetes mellitus. Diabetes Care 2010;33: Suppl 1: S62. 20042775 10.2337/dc10-S062 PMC2797383

[2] Association AD. American Diabetes Association: report of the expert committee on the diagnosis and classification of diabetes mellitus. Diabetes Care 2003;26:S5–20. 12502614 10.2337/diacare.26.2007.s5

[3] Kessous R Shoham-Vardi I Pariente G. An association between gestational diabetes mellitus and long-term maternal cardiovascular morbidity. Heart 2013;99:1118–21. 23749791 10.1136/heartjnl-2013-303945

[4] Hillier TA Pedula KL Schmidt MM. Childhood obesity and metabolic imprinting: the ongoing effects of maternal hyperglycemia. Diabetes Care 2007;30:2287–92. 17519427 10.2337/dc06-2361

[5] Bellamy L Casas J Hingorani A. Type 2 diabetes mellitus after gestational diabetes: a systematic review and meta-analysis. Lancet 2009;373:1773–9. 19465232 10.1016/S0140-6736(09)60731-5

[6] Bulletins-Obstetrics C. Practice bulletin No. 137: gestational diabetes mellitus. Obstetrics & Gynecology 2013;122:406–16. 23969827 10.1097/01.AOG.0000433006.09219.f1

[7] Thériault S Forest JC Massé J. Validation of early risk-prediction models for gestational diabetes based on clinical characteristics. Diabetes Res Clin Pract 2014;103:419–25. 24447804 10.1016/j.diabres.2013.12.009

[8] Storry JR Olsson ML. The ABO blood group system revisited: a review and update. Immunohematology 2009;25:48–59. 19927620

[9] Brian M Wolpin ATC Hartge Patricia. ABO blood group and the risk of pancreatic cancer. J Natl Cancer Inst 2009;101:424–31. 19276450 10.1093/jnci/djp020 PMC2657095

[10] Miao SY Zhou W Chen L. Influence of ABO blood group and Rhesus factor on breast cancer risk: a meta-analysis of 9665 breast cancer patients and 244,768 controls. Asia-Pac J Clin Oncol 2014;10:101–8. 23714093 10.1111/ajco.12083

[11] Li Q Yu CH Yu JH. ABO blood group and the risk of hepatocellular carcinoma: a case-control study in patients with chronic hepatitis B. PLoS one 2012;07. 10.1371/journal.pone.0029928 PMC3250489 22235351

[12] Kamil M Al-Jamal HAN Yusoff NM. Association of ABO blood groups with diabetes mellitus. Libyan J Med 2010;5: 10.3402/ljm.v3405i3400.4847. 10.3402/ljm.v5i0.4847 PMC3071167 21483592

[13] Wu O Bayoumi N Vickers MA. ABO(H) blood groups and vascular disease: a systematic review and meta-analysis. J Thromb Haemost 2010;6:62–9. 10.1111/j.1538-7836.2007.02818.x 17973651

[14] Franchini M Mengoli C Lippi G. Relationship between ABO blood group and pregnancy complications: a systematic literature analysis. Blood Transfus 2016;14:441–8. 27177402 10.2450/2016.0313-15 PMC5016304

[15] Hu FB Li TY Manson JE. Inflammatory markers and risk of developing type 2 diabetes in women. Diabetes 2004;53:693. 14988254 10.2337/diabetes.53.3.693

[16] Kiechl S Pare G Barbalic M. Association of variation at the ABO locus with circulating levels of soluble intercellular adhesion molecule-1, soluble P-selectin, and soluble E-selectin: a meta-analysis. Circ Cardiovasc Genet 1942;4:681. 10.1161/CIRCGENETICS.111.960682 PMC3278232 22010135

[17] Fagherazzi G Gusto G Clavel-Chapelon F. ABO and rhesus blood groups and risk of type 2 diabetes: evidence from the large E3N cohort study. Diabetologia 2015;58:519–22. 25533388 10.1007/s00125-014-3472-9

[18] Meo SA Rouq FA Suraya F. Association of ABO and Rh blood groups with type 2 diabetes mellitus. Eur Rev Med Pharmacol Sci 2016;20:237–42. 26875891

[19] Zhang C Li Y Wang L. Blood group AB is protective factor for gestational diabetes mellitus: a prospective population-based study in Tianjin, China. Diabetes Metab Res Rev 2015;31:627–37. 25820620 10.1002/dmrr.2650

[20] Shimodaira M Yamasaki T Nakayama T. The association of maternal ABO blood group with gestational diabetes mellitus in Japanese pregnant women. Diabetes Metab Syndr 2016;10:S102–5. 27025793 10.1016/j.dsx.2016.03.003

[21] Abdulsamet E Hatice K Ozerhan O. The role of blood groups in the development of diabetes mellitus after gestational diabetes mellitus. Ther Clin Risk Manag 2015;11:1613–7. 26527878 10.2147/TCRM.S92294 PMC4621172

[22] Phaloprakarn C Tangjitgamol S. Maternal ABO blood group and adverse pregnancy outcomes. J Perinatol 2013;33:107. 22678143 10.1038/jp.2012.73

[23] Shamseer L Moher D Clarke M. Preferred reporting items for systematic review and meta-analysis protocols (PRISMA-P) 2015: elaboration and explanation. BMJV 350 2015;g7647. 10.1136/bmj.g7647 25555855

[24] Stang A. Critical evaluation of the Newcastle – Ottawa scale for the assessment of the quality of nonrandomized studies in meta-analyses. Eur J Epidemiol 2010;25:603–5. 20652370 10.1007/s10654-010-9491-z

[25] Zhang Q Jin Y Li X. Plasminogen activator inhibitor-1 (PAI-1) 4G/5G promoter polymorphisms and risk of venous thromboembolism —- a meta-analysis and systematic review. VASA 2020;49:141–6. 31920171 10.1024/0301-1526/a000839

[26] Lewis SJ Zammit S Gunnell D. Bias in meta-analysis detected by a simple, graphical test. BMJ 1997;315:629–34. 9310563 10.1136/bmj.315.7109.629 PMC2127453

[27] Duval S Tweedie R. Trim and fill: a simple funnel-plot-based method of testing and adjusting for publication bias in meta-analysis. Biometrics 2000;56:455–63. 10877304 10.1111/j.0006-341x.2000.00455.x

[28] Kennelly MA McAuliffe FM. Prediction and prevention of gestational diabetes: an update of recent literature. Eur J Obstet Gynecol Reprod Biol 2016;202:92–8. 27235645 10.1016/j.ejogrb.2016.03.032

[29] Seyfizadeh N Seyfizadeh N Yousefi B. Is there association between ABO blood group and the risk factors of unfavorable outcomes of pregnancy? J Matern Fetal Neonatal Med 2015;28:578–82. 24849128 10.3109/14767058.2014.927424

[30] Shimodaira M Yamasaki T Nakayama T. The association of maternal ABO blood group with gestational diabetes mellitus in Japanese pregnant women. Diabetes Metab Syndr 2016;S102–5. 27025793 10.1016/j.dsx.2016.03.003

